# Altered gut microbiota in infants is associated with respiratory syncytial virus disease severity

**DOI:** 10.1186/s12866-020-01816-5

**Published:** 2020-06-01

**Authors:** Jeffrey N. Harding, David Siefker, Luan Vu, Dahui You, John DeVincenzo, JF. Pierre, Stephania A. Cormier

**Affiliations:** 1 Department of Biological Sciences, Louisiana State University, 202 Life Sciences Building, Baton Rouge, LA 70803 USA; 2 Department of Comparative Biomedical Sciences, Louisiana State University School of Veterinary Medicine, Baton Rouge, LA USA; 3 Department of Pediatrics, University of Tennessee Health Science Center, Memphis, TN USA; 4 Le Bonheur Children’s Foundation Research Center, Memphis, TN USA

**Keywords:** Respiratory syncytial virus, Human, Gut microbiome, 16S, Microbiota, Infants, Severity

## Abstract

**Background:**

Respiratory syncytial virus (RSV) is the number one cause of lower respiratory tract infections in infants. There are still no vaccines or specific antiviral therapies against RSV, mainly due to the inadequate understanding of RSV pathogenesis. Recent data suggest a role for gut microbiota community structure in determining RSV disease severity. Our objective was to determine the gut microbial profile associated with severe RSV patients, which could be used to help identify at-risk patients and develop therapeutically protective microbial assemblages that may stimulate immuno-protection.

**Results:**

We enrolled 95 infants from Le Bonheur during the 2014 to 2016 RSV season. Of these, 37 were well-babies and 58 were hospitalized with RSV. Of the RSV infected babies, 53 remained in the pediatric ward (moderate) and 5 were moved to the pediatric intensive care unit at a later date (severe). Stool samples were collected within 72 h of admission; and the composition of gut microbiota was evaluated via 16S sequencing of fecal DNA. There was a significant enrichment in S24_7, Clostridiales, Odoribacteraceae, Lactobacillaceae, and Actinomyces in RSV (moderate and severe) vs. controls. Patients with severe RSV disease had slightly lower alpha diversity (richness and evenness of the bacterial community) of the gut microbiota compared to patients with moderate RSV and healthy controls. Beta diversity (overall microbial composition) was significantly different between all RSV patients (moderate and severe) compared to controls and had significant microbial composition separating all three groups (control, moderate RSV, and severe RSV).

**Conclusions:**

Collectively, these data demonstrate that a unique gut microbial profile is associated with RSV disease and with severe RSV disease with admission to the pediatric intensive care unit. More mechanistic experiments are needed to determine whether the differences observed in gut microbiota are the cause or consequences of severe RSV disease.

## Background

Respiratory syncytial virus (RSV) is a ubiquitous respiratory virus infecting the majority of the human population by 1 year of age [1]. While most infants develop mild upper respiratory tract infections (URTI), 0.5–2% develop severe, lower respiratory tract infections (LRTI), including bronchiolitis and pneumonia, requiring hospitalization [2]. Each year, RSV causes 33 million cases and 3.4 million hospitalizations worldwide in children under 5 years of age [3]. In the U.S. alone, RSV causes 85,000 to 144,000 hospitalizations [4], 14,000 deaths [2], and ~ $2.6 billion in medical care costs each year [5]. Despite the substantial RSV-associated medical and economic burden worldwide, a vaccine for RSV currently remains elusive. The only prevention for infants at high-risk for complications (i.e., premature or underlying immune/cardiopulmonary diseases) is in the form of a monoclonal antibody, palivizumab. In view of the fact that RSV is a “significant unmet medical need” [6] with no known vaccine, there is a necessity to find therapeutic approaches to prevent and treat severe RSV infections.

The recently discovered relationship between respiratory health and gut microbiome dysbiosis has attracted growing interest for therapeutic manipulation to improve vaccine effectiveness and to allow for alternative therapies for viruses with no known vaccines, like RSV. Clinical studies demonstrate that prebiotic and probiotic supplementation alters gut microbiota [7], reduces the incidence of rhinovirus infections in preterm infants [8], and reduces rhinovirus infection duration and severity in adults [9]. In mice, gut microbial dysbiosis induced by antibiotic treatment inhibits pulmonary type I interferons (IFNs) and T cell responses, resulting in increased lung viral loads after infection with influenza virus [10, 11]. On the other hand, supplementing mice with *Lactobacillus plantarum* promotes type I IFNs and reduces influenza viral load in the lung [12]. In mice, both RSV and influenza virus infection alters the gut microbiome and provides preferential growth environments for the *S24_7* family showing a correlation between *S24_7* family and RSV infection, although the mechanism is still unknown [13]. Several reports have studied the association of airway microbiota with RSV severity [14, 15]; nevertheless, there have been no studies that directly investigated the role of gut microbiota in the severity of RSV infections.

We hypothesize that gut microbiota could play an essential role in the pathogenesis of RSV in infants. This study characterizes the gut microbiome in infants hospitalized with RSV infection differentiating severe infections requiring infants to be put into pediatric intensive care unit (PICU) and moderate infections of infants in the general ward. Our results demonstrate that there is a relationship between RSV infection and gut microbiome that could conceivably be interrogated to develop a useful therapeutic target to prevent severe RSV disease.

## Results

### Characteristics of study population

Our study population included 37 patients enrolled from Le Bonheur Outpatient Clinic during well-baby checkup (control) and 58 patients admitted to the general ward (moderate), who tested positive for RSV and negative for influenza by RT-PCR. Samples were collected from patients during the 2014 to 2016 RSV seasons. Subsequent to enrollment, 5 patients were moved from the general ward to the pediatric intensive care unit (PICU). These patients were classified as severe. None of the enrolled patients received antibiotics prior to sample collection. Control, moderate, and severe patients had an average age of 93, 94, and 60 days, respectively (*p* = 0.284; Table 1). The majority of moderate and severe patients had O2 supplementation (*p* = < 0.001) and wheezing (*p* = 0.011) compared to the control group. From patient enrollment forms, there were no significant differences in the average time of symptom onset in moderate patients (6.82 days) compared to severe patients (7.33 days). As both wheezing and O2 supplementation is commonly associated with RSV, this correlation is not surprising. However, the only significant difference observed between the RSV groups (moderate and severe) regarding all demographic metadata was in the family history of asthma due to none of the severe patients having a family history of asthma (Table 1).

**Table 1.**
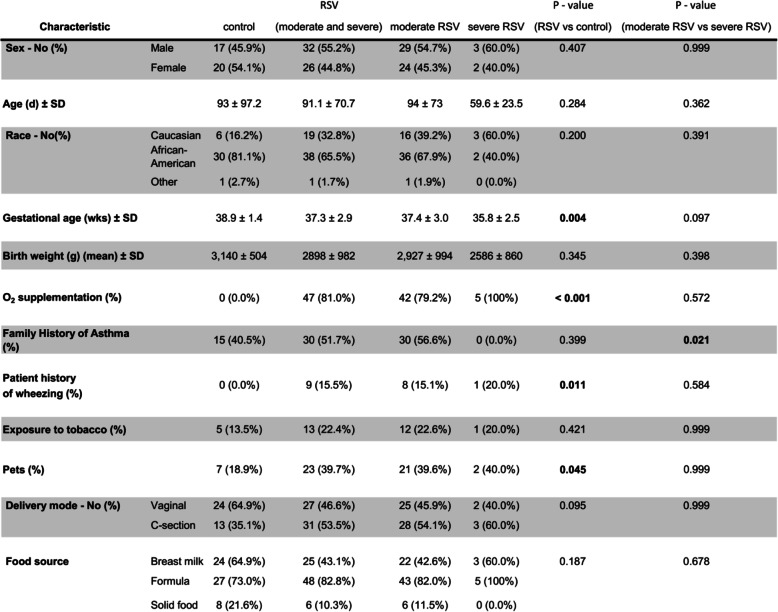
Participant characteristics of healthy controls and infants hospitalized with RSV

*P* values in the table were calculated using Fisher’s Exact test

### The gut microbiome

**Fig. 1:**
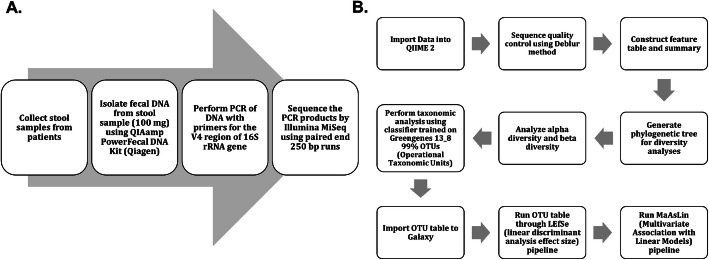
Sample collection process and data analysis workflow. **a** Workflow from sample collection to sequence data. **b** Data analysis workflow in Qiime 2 and Galaxy

Although the role of gut microbiota in regulating the immune system and respiratory infections is being increasingly recognized, the role of gut microbiota in RSV disease severity has never been addressed in infant humans. Here, we analyzed the gut microbiota from infants hospitalized with RSV. The patient fecal DNA was isolated for microbiome analysis by 16S rRNA sequencing (Fig. 1a). Resulting sequencing data was analyzed using Qiime 2 pipeline with a 99% OTU identification by GreenGenes database (Fig. 1b).

### Gut microbiome richness is reduced in severe RSV infected patients

**Fig. 2:**
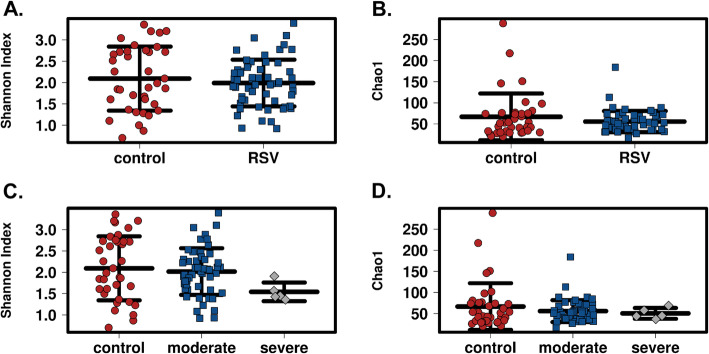
α-diversity is not significantly reduced in severe RSV infected patients compared to healthy controls. **a** Shannon Index of the gut microbiota shows no difference between healthy controls (*n* = 37) and infants with RSV disease (*n* = 58). **b** Chao1 Index of the gut microbiota shows no difference between healthy controls (*n* = 37) and infants with RSV disease (*n* = 58). **c** Shannon Index is slightly reduced in severe RSV disease (*n* = 5) compared to healthy controls (*n* = 37) or infants with moderate RSV disease (*n* = 53) but is not significant. **d** Chao1 Index shows no difference in severe RSV disease (*n* = 5) compared to healthy controls (*n* = 37) and infants with moderate RSV disease (*n* = 53). Each point represents an individual infant and the mean ± SEM

To examine the gut microbiota in RSV patients, we determined the α-diversity in the stool samples using Chao1 index and Shannon index in QIIME 2 with samples rarefied to a read depth of 6682 to ensure that a reasonable number of sequence reads have been obtained for each OTU (Supplemental Figure 1A). There was no difference in the species richness and evenness (α-diversity) of gut microbiome between RSV patients and control patients (Fig. 2a, b). We further segregated the RSV patients into moderate and severe RSV patients (based on their PICU status) to determine if there are differences in gut microbiome as a result of severity of RSV disease. Although it failed to reach significance, patients with severe RSV (PICU) had a slightly reduced species richness and evenness in the gut microbiota compared to that of control and moderate RSV (non-PICU) patients using both Shannon index and Chao1 index (Fig. 2c, d).

### Gut microbiome phylogenetic diversity shows significant clustered populations in RSV disease severity

**Fig. 3:**
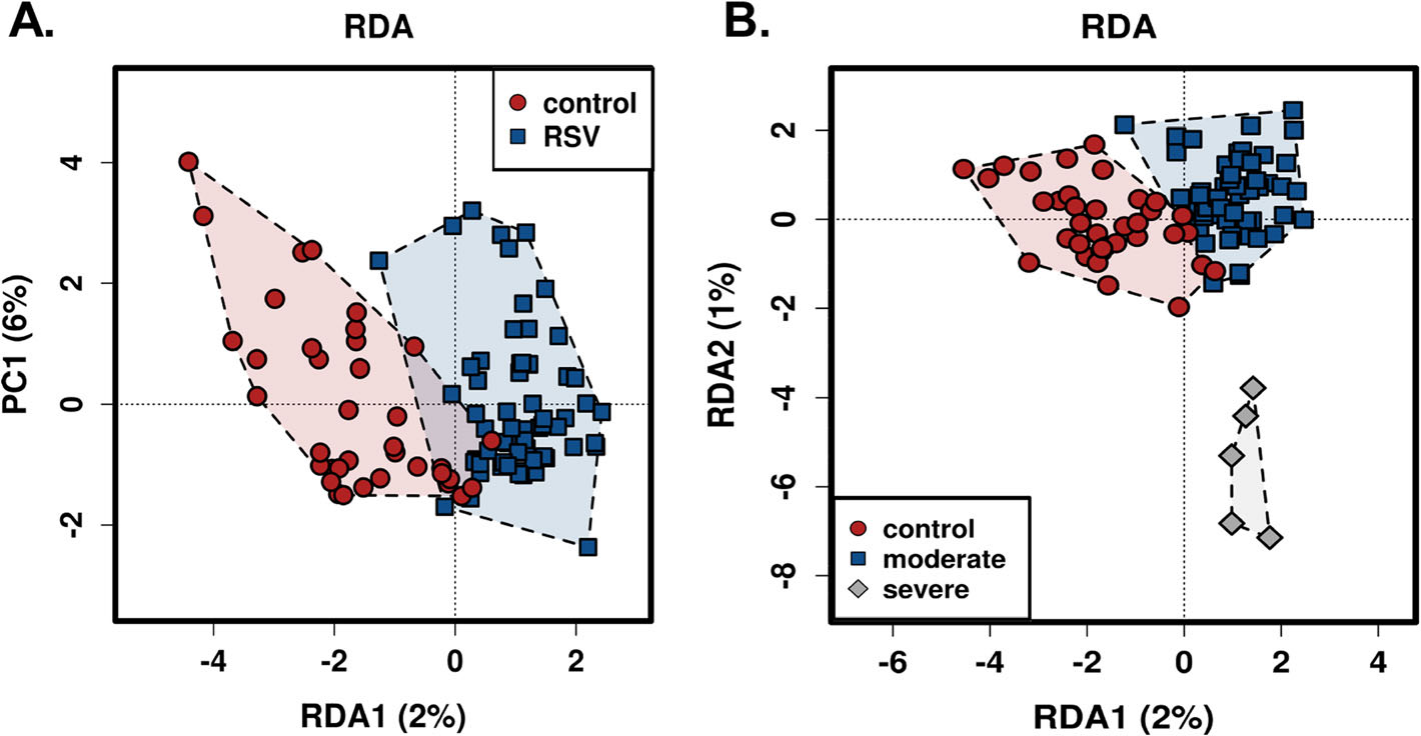
RDA plots show separate phylogenetic clustering for control and RSV patients. Gut microbiome composition in infants infected with RSV and healthy control infants were analyzed using RDA to visualize the phylogenetic dissociations in RSV infected patients compared to healthy controls. **a** Control patients cluster separately from moderate or severe RSV patients on a phylogenetic basis. **b** Severe RSV patients cluster separately from control and moderate RSV patients showing a significantly different phylogenetic composition

**Fig. 4:**
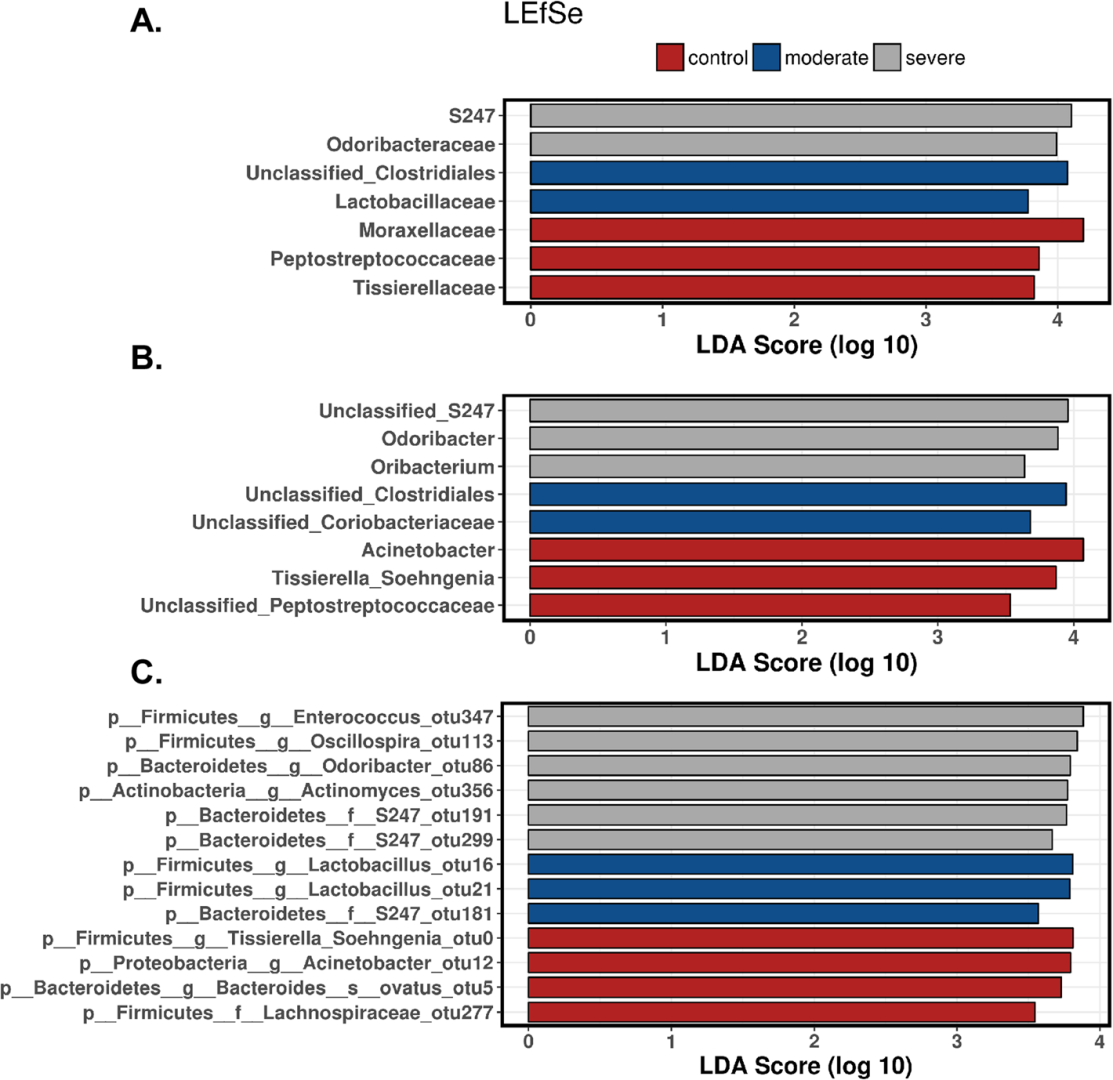
Linear discriminant analysis effect size (LEfSe) analysis of severe, moderate, and control shows six specific bacterial clades that distinguish severe RSV from control or moderate RSV. The LEfSe analysis shows the linear effect size between the samples and calculates the linear discriminant analysis (LDA) score for each of the Operation Taxonomic Units (OTUs) showing their respective ability to characterize the severity of disease. Taxonomic rank is denoted by the first small letter in the naming. **a** LEfSe at the family level, (**b**) LEfSe at the genus level, and (**c**) LEfSe at the OTU level. All the phylogenetic clades shown are *p* < 0.05 following non-parametric factorial Kruskal-Wallis (KW) sum-rank test and Wilcoxon rank-sum test

**Fig. 5:**
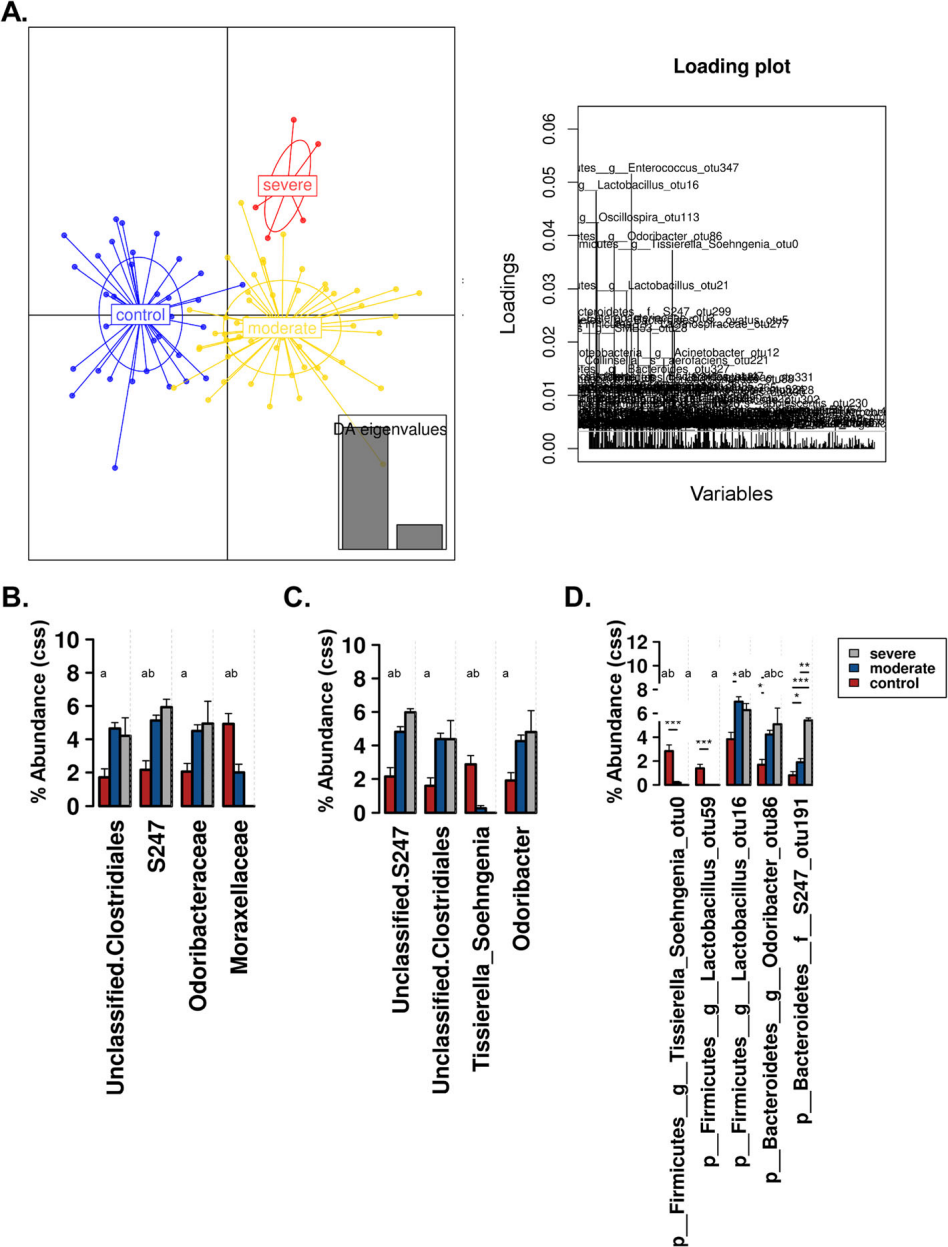
Discriminant analysis of principal components (DAPC) plot (**a**) at OTU level revealed distinct clustering of severe patients (red), moderate patients (yellow), and control patients (blue). Canonical loading plot stresses the specific OTUs most influential in the separation of clusters. **b** Percent abundance with cumulative-sum scaling (CSS) + log transformation at the family level. **c** Percent abundance with CSS + log transformation at the genus level. **d** Percent abundance with CSS + log transformation at the OTU level. Significance (*p* < 0.05) with BH correction between control vs. moderate, control vs. severe, and moderate vs. severe is denoted by a, b, and c, respectively

In addition to richness and evenness of gut microbiota, we determined the overall qualitative relatedness of phylogenetic distances (β-diversity). Significant differences were found in microbiome composition between RSV and control group as well as between RSV disease severities. We applied the multivariate method of PERMANOVA with 999 permutations using unweighted and weighted UniFrac distance matrix (*P* < 0.0001 and *P* = 0.031, respectively). In addition, we used multivariate redundant discriminant analysis (RDA) at the OTU level to visualize the separation of RSV and the control groups (Fig. 3a). The patients were further distinguished into groups by disease severity (Fig. 3b). This showed that there were not only statistically significant differences between the control and RSV patients, but there was a further separation between severity of RSV in the patients (*P* < 0.05). To expand our understanding of the phylogenetic differences driving the separation of clusters, we performed Linear Discriminate Analysis of Effect size (LEfSe) analysis to identify enriched taxa that best characterize the alterations in the severe, moderate, and control patients by coupling standard tests for statistical significance and biological relevance through prior knowledge stored in databases such as KEGG, SEED, COGs, etc.. We were able to identify the key phylotypes that could be used as biomarkers at different phylogenetic levels to discriminate between control, moderate, and severe RSV (Fig. 4; Supplemental Figures 2 and 3). LEfSe comparison of the gut microbiota highlights the distinctive microbial profile of patients with severe disease. The taxa in gray show the specific microbial groups that are enriched in the severe patients when comparing to the moderate and control groups. The blue and the red colors show the specific microbial groups that are enriched in the microbiome for moderate and control patients, respectively. Due to the fact that LEfSe includes biologically informative clades differentiating two or more phenotypes, this may be important for clinical therapeutics. On a family level we see that *S24_7* and *Odoribacteraceae* are associated with severe RSV, while *Clostridiales* and *Lactobacillaceae* are characteristic of moderate RSV (Fig. 4a). At the genus level, we observed *S24_7*, *Odoribacter*, and *Oribacterium* being associated with severe RSV and *Clostridiales* and *Coriobacteriaceae* being associated with moderate disease (Fig. 4b). The specific microbes associated with genus and OTUs are also shown in Fig. 4c. Furthermore, we used discriminant analysis of principal components at the OTU level in conjunction with one-way ANOVA with Benjamini-Hochberg correction and Tukey’s range test to further elucidate the microbial differences between disease severity groups. We showed that specific OTUs belonging to the *Enterococcus*, *Lactobacillus*, *Oscillospira*, *Odoribacter*, *Tissierella Soehngenia*, and *S24_7* families were among the top bacterial taxa to separate the specific microbiome characteristics between the groups in a discriminant analysis of principal components (DAPC) cluster graph (Fig. 5a). DAPC analysis utilizes sequential K-means and model selection to infer genetic clusters by focusing on between-group variability and identifying the variables that best separate these groups. This is further demonstrated by looking at the disease severity groups at the Family level. By inspecting the percent abundance of the families demonstrated to separate the disease severity groups, *S24_7* is increased in severe patients compared to control patients while *Moraxellaceae* is decreased (Fig. 5b). At the genus level, *S24_7* is again increased in severe patients compared to control patients while *Tissierella Soehngenia* is decreased (Fig. 5c). At the OTU level, we observed an increase in relative percent abundance of *S24_7 and Odoribacter* with a decrease in *Tissierella Soehngenia*, between severe and control RSV (Fig. 5d). However, the most significant of these was significant increases in *S24_7* in severe patients compared to moderate patients. This coincides with the LEfSe analysis, giving additional data showing that there is an association with *S24_7* and severity of RSV disease.

## Discussion

The lung and gut microbiome axis has increasingly been studied over the last decade to elucidate the relationship between microbiome dysbiosis and immunity in the lung [16–22]. The gut-lung microbiome axis has been proposed and studied for its ability to help regulate the immune system [13, 22, 23]. Because there are a vast number of microbes residing in the gut, there is no shortage of microorganism-associated molecular patterns (MAMPs) as well as pathogen-associated molecular patterns (PAMPs) that can initiate in the gut and alter immune functions in the lung [24]. MAMPs and PAMPS are able to activate Toll Like Receptors (TLR) on dendritic cells, T cells, epithelial cells, macrophages, and B cells. This activation of TLRs can change cytokine, chemokine, and antibody production in the lung, all of which have been shown to be important in viral clearance for RSV [25, 26]. Studies have shown associations between bronchiolitis and the gut microbiome in infants, along with associations between RSV and the gut microbiome of mice. In infants, these studies demonstrated that there was a Bacteroides-dominant profile that was associated with a higher likelihood of bronchiolitis [27]. In mice, there was a significant increase in Bacteroidetes and a decrease in Firmicutes phyla abundance [13]. However, to our knowledge, our study is the first to directly investigate the role of gut microbiota in RSV disease severity in infants. Our findings indicate that between RSV infected patients and control patients there are no significant changes in the abundance of microbes in the gut (Fig. 2a, b**)**, but there is a significant change in gut microbial composition between RSV infected patients and control patients (Fig. 3a).

We observed significant enrichments in the families *S24_7*, *Clostridiales*, *Odoribacteraceae*, *Lactobacillaceae*, *Actinomyces* using LEfSe and ANOVA with BH correction between these RSV and control patients. Likewise, our data indicates that when looking at severity (PICU vs. Non-PICU vs. Control), there is only a slight decrease in commensal microbial abundance across the patient groups (Fig. 2c, d). We do, however, see separate phylogenetic clustering between the severe and moderate patients and separation from the control patients (Fig. 3b). This clustering was shown to be dependent upon *Enterococcus*, *Lactobacillus*, *Oscillospira*, *Odoribacter*, *Tissierella Soehngenia*, and *S24_7* (Fig. 4). The LEfSe analysis showed us 6 bacterial OTUs that were enriched in the severe group when compared to the moderate and control groups. Specifically, a significant increase in S24_7 OTU 191 coincided with severe versus moderate RSV (Fig. 5d). This data suggests that the bacteria that are driving these differences are encompassed within the Firmicutes and Bacteroidetes phylum but are varied at the family and OTU levels illustrating unknown factors contributing to this dysbiosis of the gut microbiome. Our study demonstrates that the gut microbiome diversification is associated with RSV disease severity and suggests that altering the gut microbiome may have clinical relevance.

Despite the fact that *S24_7* (aka *Muribaculaceae* or *Candidatus Homeothermaceae*) has been shown to make up a significant portion of the mouse and human intestinal microbiome, there have been very few studies investigating its role in immunity [13, 28, 29]. One study characterizing 30 population genomes of *S24_7* found that 20 of the 30 populations’ genomes contain a metalloprotease belonging to M6 peptidase family. Peptidases within this family exhibit antimicrobial capabilities as well as degradation abilities for extracellular matrices (ECM). In addition to M6 peptidase in their genomes, 11 of the 30 populations of *S24_7* contained IgA degrading peptidase sequences in their genomes (peptidase family M64). This could be crucial to the microbiome’s interaction with RSV severity as it has been shown that IgA is vital for mucosal defense in RSV infection and aids in protection to upper respiratory tract infections [30–34]. These mucosal antibodies have, similarly, been shown to protect human adults from experimental RSV infection [30, 35]. Therefore, IgA is important for protection and disruption of memory IgA can contribute to severity and reoccurrence of RSV.

However, *S24_7* is not the only potential mechanism for IgA disruption originating in the gut microbiome. The gut microbiome consists of thousands of commensal bacteria, all of which, play an essential role in immune response and development at an early age. The gut microbiome produces several metabolites that enter the intestinal lymphatic system allowing commensal bacteria in the gut to modulate activation of B cells, dendritic cells [36], inflammatory cytokines, and production of antibodies. Work in adult germ-free mice have demonstrated that a lack of commensal bacteria inhabiting the gut leads to fewer intraepithelial lymphocytes, smaller peyer’s patches [37], fewer Th17 T cells [38], and defective regulatory T cells [39]. In addition, human gut bacteria such as *Bacteroides fragilis* have been shown to regulate the balance between Th1 and Th2 cells in the lung by producing polysaccharide A and inducing INF-ɣ (Th1 T cells) [40]. While *Clostridium* and *Clostridiales* have been shown to induce regulatory T cell (Treg) production [41]. In RSV infected patients, we observed increases in the *Clostridiales* family suggesting an induction of Tregs which are essential in the management of RSV severity. As RSV evades the adaptive immune response by skewing the Th1/Th2 balances toward a Th2 specific immune response, the balance between Th1, Th2, and Treg cells are crucial to the pathogenesis of RSV by reducing Th2 immune responses and pulmonary eosinophilia [42]. Therefore, the microbiome dysbiosis that we observe could be a source for T cell imbalance and severe RSV disease. While it is enticing to speculate that elevations in M6 peptidases due to enrichment of S24_7 family members leads to degradation of RSV specific IgA and disease severity, it is yet to be determined whether a gut microbial profile low in diversity and enriched in *S24_7* is the cause, or result, of severe RSV disease. Mechanistic studies need to be done in order to address this important question. It is possible that by elucidating the mechanism of *S24_7* in RSV disease severity therapeutic targets could be identified.

Our findings highlight that there is a correlation between RSV infection and dysbiosis of the gut microbiome. We identified disruptions in the abundance of microbes in patients with severe RSV disease as well as characteristic microbiome shifts in all RSV patients. We identified that there were 6 specific, enriched microbes associated with RSV severity. Together, our findings identified changes in phylogenetic diversity in RSV patients and identified specific microbes associated with severity of disease. However, there were limitations to this study. One of the limitations of this study is the small sample size, particularly for severe RSV patients. It is also noteworthy that this study was conducted in Memphis, Tennessee where there is a socioeconomic and racial skew in patient demographics that can be a limitation to the extrapolation of these studies across sites. It is unclear if the changes in gut microbiota are causal or correlated with RSV (or hospitalization). Further studies need to be done to characterize the change in gut microbiota of RSV patients fully. Future work will include additional patient recruitment to more precisely define a gut microbial profile associated with severe RSV disease. These studies could lead to the development of logistic regression models to predict infants at high risk for severe RSV disease based on gut microbiome.

## Conclusions

Respiratory syncytial virus (RSV) is a ubiquitous respiratory virus infecting the majority of the human population by 1 year of age. It is the number one cause of lower respiratory tract infections in infants, yet there are still no vaccines or specific antiviral therapies against RSV. Most patients infected with RSV develop mild upper respiratory tract infections, but a percentage of patients develop severe lower respiratory tract infections eliciting the need for intensive care and sometimes resulting in death. While there have been papers that show the interrelationships between gut and nasal microbiome changes in RSV patients there has been no data that observes the relationship between gut microbiota and severity of RSV disease. This manuscript makes the case that gut microbiome changes are associated with severity of RSV disease and these changes could be used to identify therapeutic targets or as biomarkers of severe clinical RSV. The specific interaction of the gut microbiome and respiratory immunity against RSV infections should be investigated in further studies.

## Methods

### Ethics statement

These studies were approved by the Institutional Review Board at the University of Tennessee Health Science Center and Louisiana State University.

### Patient enrollment and sample collection

Patients were enrolled at Le Bonheur Children’s Hospital in Memphis, Tennessee, during the RSV season in the years 2014–2016. A total of 58 infants hospitalized with RSV were enrolled in the study (Table 1). This work includes samples from 23 patients that were used in a study recently submitted for publication and 33 patients used in another study recently accepted for publication [43]. Inclusion criteria were patients less than 12 months old (1 control patient was 368 days old), informed consent from parent or guardian, patients could not have a positive blood culture within 72 h prior to collection of stool, patients could not be diagnosed with any immunodeficiency, patients could not be on any antibiotics within 4 weeks prior to enrollment, patients could not have been placed on oxygen for more than 7 days within 3 months prior to the study, patients could not be receiving other investigational immunomodulatory or investigational antiviral agents, patients could not have a hemodynamically significant congenital heart disease, and patients must have tested positive for RSV and negative for influenza, by RT-PCR or be positive for RSV antigens tested by the hospital’s diagnostic lab. Among RSV positive patients, 5 infants were admitted to the PICU and thus considered severe; 53 were admitted to the pediatric ward and considered moderate. Healthy controls patients were enrolled from Le Bonheur Outpatient Clinic during well-baby checkups. Within 72 h of admission to the hospital, stool samples from these infants were collected, and 100 mg aliquots of stool were stored at − 80 °C until DNA extraction.

### 16S sequencing

Fecal DNA was isolated from the 100 mg aliquots of stool using QIAamp PowerFecal DNA Kit (Qiagen) according to the manufacturer’s instructions. Fecal DNA was sent to the University of Alabama at Birmingham Center for Clinical and Translational Science for 16S sequencing. Unique barcoded primers were used to amplify the hypervariable V4 regions of the bacterial 16S rRNA gene from each sample. The resulting amplicon libraries were then gel purified using QIAquick Gel Extraction Kit (Qiagen). Finally, the purified libraries were sequenced using Next-generation sequencing performed on the Illumina MiSeq system using 250 bp paired-end reads (Fig. 1a). All generation of amplicon libraries and sample preparation for loadings onto the Illumina MiSeq system protocols were previously published by Kumar et al. [44]

### Bioinformatics

Sequence data with a minimum length of 250 base pairs were processed and analyzed using QIIME 2 version 2018.4.0 (RRID: SCR_008249). Sequences were demultiplexed using Qiime 2 demux, passed through quality control using the deblur quality control method, and created the phylogenetic tree using mafft program to perform a multiple sequence alignment of the sequences and FastTree to create an unrooted tree. The operational taxonomic units (OTUs) were generated using a 99% identity and then classified by phylogeny using the classifier from GreenGenes database 13_8 [45, 46]. Subsequently, alpha diversity measured using Shannon Index and Chao1 Index in Qiime2, beta diversity, the community similarity, measured via unweighted and weighted unifrac distance matrix and PERMANOVA with 999 permutations was used to identify significant features between groups. Linear discriminant analysis effect size (LEfSe: RRID: SCR_014609) in Galaxy (Fig. 1b) was used to discover biomarkers specific to each of the patient groups. In Calypso, the data was normalized using cumulative sum scaling (CSS) normalization for multivariate tests. In order to address false discovery rate two-way repeated-measures analysis of variance (ANOVA) in conjunction with Benjamini–Hochberg multiple-inference correction with Dunnett’s correction was used to test for significant differences in alpha diversity and differential taxa abundance between groups. Significance was defined as *p* < 0.05 after FDR adjustment (Prism v8.0, GraphPad Software, La Jolla, CA) (Calypso). Quality control of sample data can be found in (Supplemental Figure 1). Data are presented as means ± SEM. The datasets generated for this study can be found in the SRA accession database: PRJNA579491.

## Supplementary information

**Additional file 1: Figure S1.** Quality control plots **(A)** rarefaction analysis showing adequate reads for OTUs. **(B)** reads per sample showing high read counts for all samples used.

**Additional file 2: Figure S2.** Bar chart illustrating the microbial abundance differences between the control, moderate, and severe patients at the **(A)** family level, **(B)** genus level, and **(C)** OTU level.

**Additional file 3:Figure S3.** Odds ratio showing the top 100 biomarkers associated with all RSV patients compared to control patients.

## Data Availability

The datasets generated for this study can be found in the SRA accession database: PRJNA579491. https://www.ncbi.nlm.nih.gov/Traces/study/?acc=PRJNA579491&o=acc_s%3Aa

## Abbreviations

RSV: Respiratory syncytial virus
URTI: Upper respiratory tract infections
LRTI: Lower respiratory tract infections
PICU: Pediatric intensive care unit
OTU: Operational taxonomic units
LEfSe: Linear discriminant analysis effect size
LDA: Linear discriminant analysis
CSS: Cumulative sum scaling
RDA: Redundant discriminant analysis
DAPC: Discriminant analysis of principal components
PAMP: Pathogen-associated molecular patterns
TLR: Toll Like Receptors
