# Supplementary figures and images for: Altered gut microbiota in infants is associated with respiratory syncytial virus disease severity
Source: BMC Microbiol. 2020 Jun 1;20:140. doi: 10.1186/s12866-020-01816-5 (PMC7268675; doi:10.1186/s12866-020-01816-5)

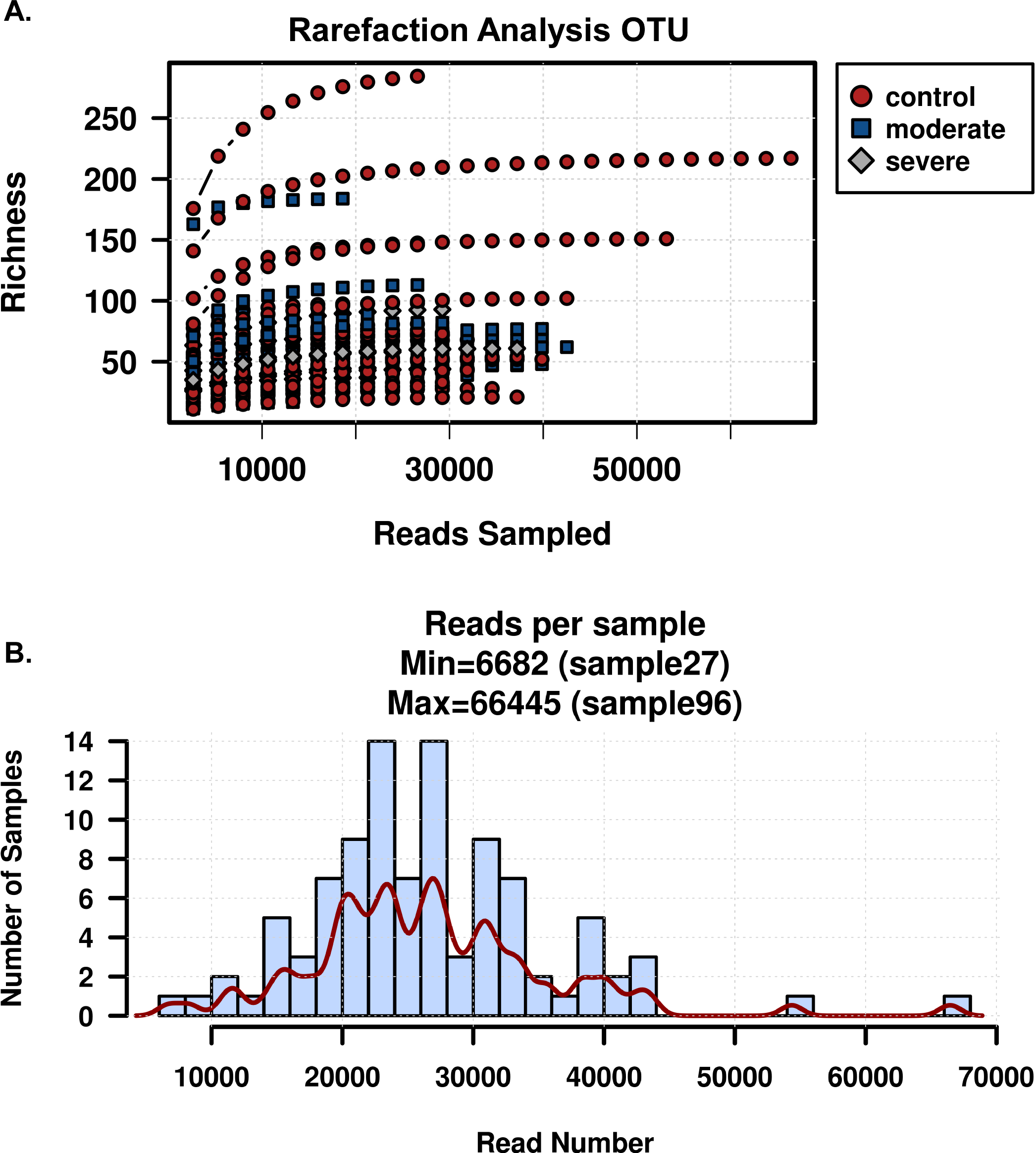

Supplement: Supplementary file 1 — Additional file 1: Figure S1. Quality control plots (A) rarefaction analysis showing adequate reads for OTUs. (B) reads per sample showing high read counts for all samples used. [file 12866_2020_1816_MOESM1_ESM.tif]

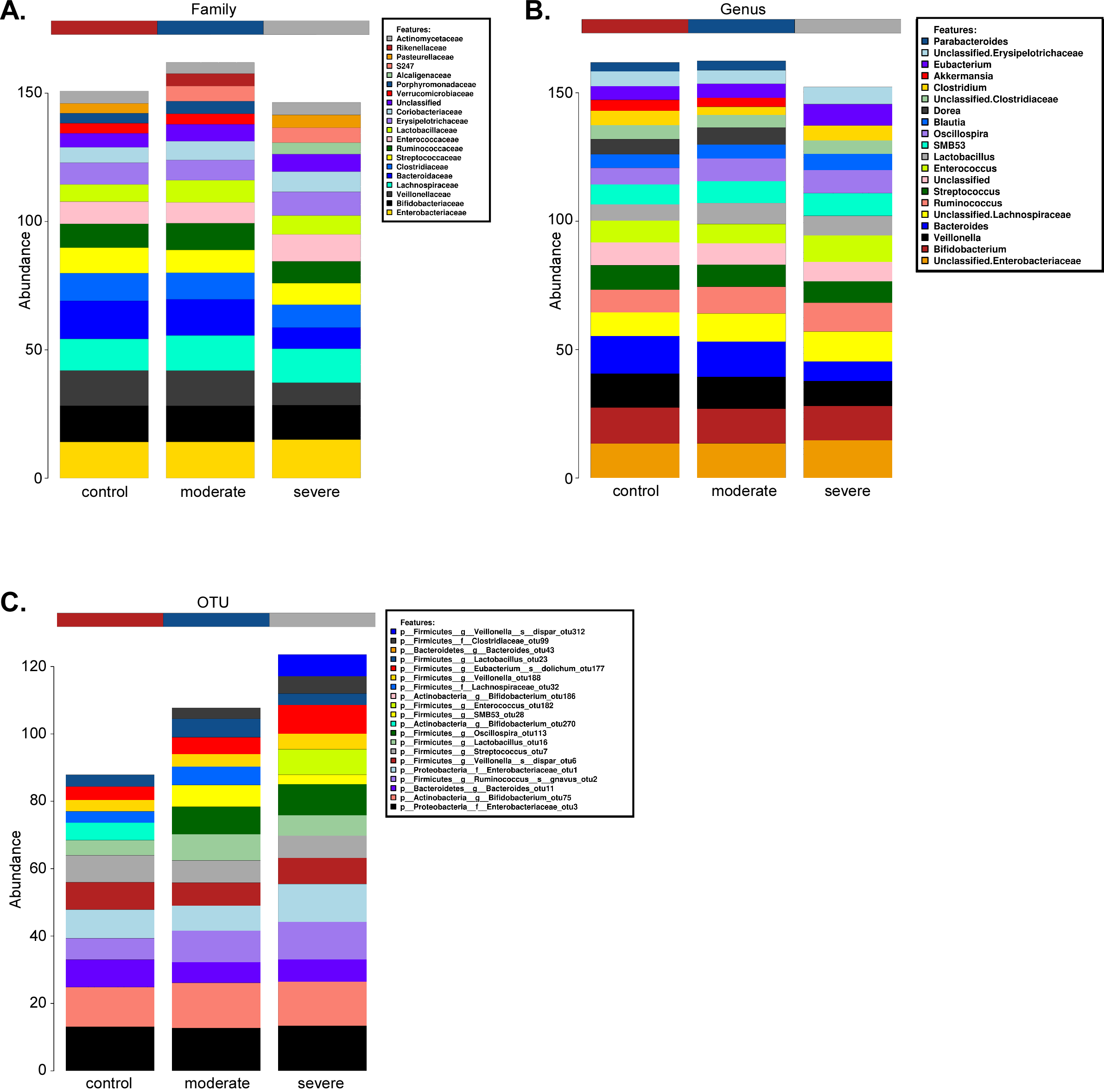

Supplement: Supplementary file 2 — Additional file 2: Figure S2. Bar chart illustrating the microbial abundance differences between the control, moderate, and severe patients at the (A) family level, (B) genus level, and (C) OTU level. [file 12866_2020_1816_MOESM2_ESM.tif]

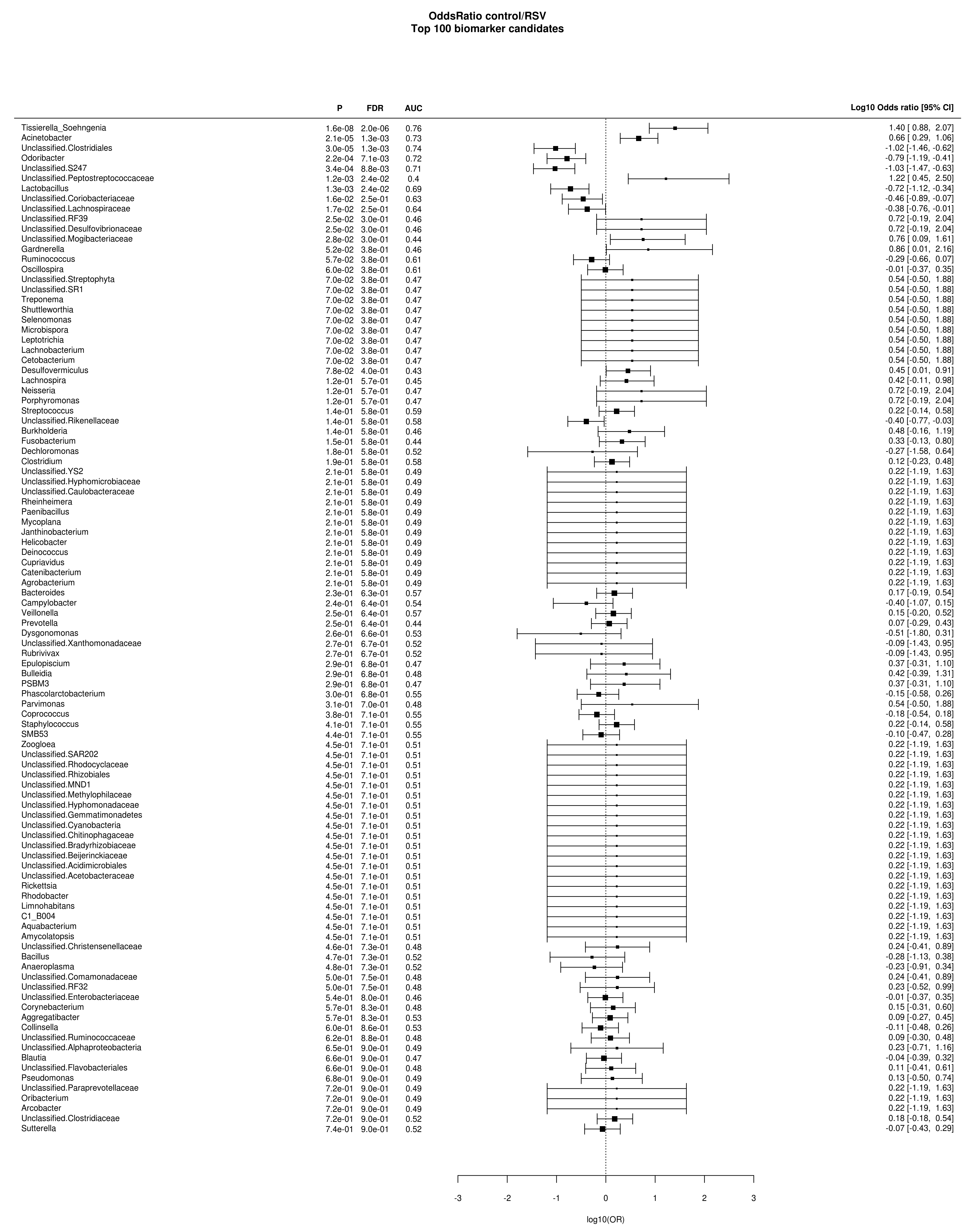

Supplement: Supplementary file 3 — Additional file 3:Figure S3. Odds ratio showing the top 100 biomarkers associated with all RSV patients compared to control patients. [file 12866_2020_1816_MOESM3_ESM.tif]
